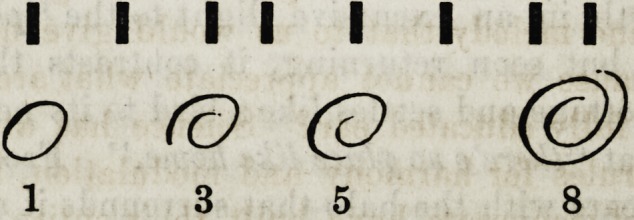# Paper Read before the New Orleans Academy of Sciences

**Published:** 1859-10

**Authors:** John S. Clark


### ARTICLE IV.

*Paper read before the New Orleans Academy of Sciences.* By JOHN S. CLARK, D. D. S., Fellow of the Academy, and published in this Journal by permission.

### *Empiricism in Science.*

THERE are two ways of conducting scientific investigation. The *empirical* or purely *physical*, and the metaphysical. The empirical consists in demanding an open record of facts and experiences, that by experiment prove by factitious analysis and synthesis not only tangible to all who have the industry to examine them, but, it contracts the record to the narrow limit of human understanding and solution. It rejects all hypotheses that do not come within the range of actual experience.

It ignores faith, (in its secular sense belief in what we cannot understand,) and while it leads us forth into the wide realm of nature by heaven-borrowed light, it denies its agency in illuminating the gems of truth that flash upon its pathway, counting them *sources* instead of reflectors of light.

The other method is simply the study of *science illumi-*

*nated.* It recognizes in nature a language of design, and discovers nothing in science but traces of a Creator's hand.

It adjusts the facts and principles open to finite reason in their proper order of solution, but its philosophy looks beyond mere isolated facts for a more comprehensive reason for that adjustment.

Suppose two students in science together trace physiologically the history of animated nature as it descends from man down to the lowest order of animal life, until it is lost in its undefined blending into the vegetable kingdom. One of them with the cry of "Eureka," "I have found it," attempts to demonstrate that from the earth at the point of union between the animal and vegetable kingdom, spring spontaneously both of those series. And as we know that in the world's history these productions have been in regular progressive series from the lower to the higher, so man has been a production of that series from the earth's spontaneous birth.

But, says the other, admitting all this to be true, who told you all this? With what light and by what power did you discover your noble progenitors? When, how, and where did you receive into your insecto-animal nature, this power of reasoning so logically? "Science (says the first) is '*to know,*' and as we *do not know*, we have nothing to do with that we cannot understand." "Science calls on us for facts which we can demonstrate, and she will know nothing beyond the exact record." "In animated nature we reason by analogy, but the likeness must be within our experience, as we have no standard higher than we are acquainted with."

In the world of inanimate matter we take a substance and by analogy we classify it, by analysis (taking it apart) and by synthesis (putting it together) we know its constituents.

Thus science obtains its exact results, nor deals with ought beside. It is not the design of this paper to discuss any particular theory advanced by any one, but the object sought is the *true system* of examination.

In a former paper which I had the honor to read before the Academy, it was assumed that faith (in its secular sense) is necessary at every step we take in scientific investigation. Also as a natural inference from the nature of science as there stated, the full and complete development of any science can never be attained by human intellect.

The denial to the records of science of that mysterious and to us incomprehensible substratum on which it must ever rely for material with which to construct its philosophy, is the grand error into which the empirical reasoner falls. It is a natural error. For it does not comport with the proud heart of man to remind him that he is a very atom in the vast universe of nature.

That all the mighty achievements of his life that seem to him so great, are but the insect struggle that flaps its tiny wing in the morning sun—dependent on tempered winds as it floats along to the close of day and of life.

But such even science will tell us, is the stern, rigid truth—disguise it as we may, and there is but one difference between the two. The insect acts by instinct, the man by reason. Or simply the one has an eye that reflects only the objects in the immediate field of vision, while the other with telescopic range, looks far away into the vaster fields of the universe, and calls that knowledge which it sees—because faith surmounts the giddy heights and penetrates the dark abysses that lie in its aerial pathway. The one glues its little thread in some sequestered nook, and by sight sails across the measured space.

The other, with his line of light fast anchored in its *great source* swings across mysterious depths, catching the golden threads of truth, down which glide angel-thoughts. The animal measures that which it can see, actual contact forming the limit of its power. Man measures and weighs the tangible and intangible by powers, rules and scales, the very adjustment of which he does not understand, but in which his earnest belief causes him to place implicit reliance.

What is this great philosopher's stone of reason, this universal solvent, the mind? What are its powers, where its limit? If we say that it can fully comprehend any of the great systems of natural laws, we err. If we say we will not accept as truths the incomprehensible treasures it brings, we err and do violence to our consciousness.

This, to us, great world, as it flies round the sun excites no special wonder, for our faith endorses it, and we are sure we understand some of the laws of its motion. But must we not stop here with our *certain science?* Can we say that because we see that its diurnal velocity of revolving motion acts harmoniously with its annual circuit, our knowledge is sufficient to solve the whole problem? Is it not better to recognize the fact that this little moat floating in the sunbeam of Omnipotence, was mysteriously led forth, and to this hour is cradled in the *hand of mysterious* power? Because we understand a few of the laws of motion are we to deny the plainer facts that underlie the the whole?

So in regard to reason and the mind; we know some of the laws of computation, reason from cause to effect, and have gathered some principles into a harmonious circle, which we call science. But by what power? By the power of a single ray from the great sun, we discover the very components of the atmosphere we breathe. We can tell how much oxygen and hydrogen it contains. But when it sends forth its wingless messenger through planetary atmospheres, and beyond suns and systems, how much can we tell of the ethereal essence in which it floats? and still we believe the facts it collects in its heavenward mission.

Can it be the teaching of true philosophy, that while it cannot take a step toward solution without accepting as fact, that which is incomprehensible, still pronounces all that cannot be measured, weighed, or analyzed, a myth, a vagary of the brain, fit only for schoolmen fanatics, or imbecile? With what consistency can men of science form conclusions and give us principles founded entirely on belief, and still deny that faith in them is not an essential element?

The simplest act we put forth will show it. Science tells us to take this substance which contains oxygen, and put it in contact with that alkali, and a salt is obtained. We perform the experiment. Science is satisfied, and writes down the result. But what is demonstrated here?

Ask the purely experimental reasoner to give us the philosophy of that motion. Perhaps he will tell us of the brain and nerves acting on the muscles. How very clear that makes it. That mysterious hand, that servant of the will, is demonstration. That hand, whose movement at the will's command is as mysterious as that imponderable electric pulse of nature that thrills with the speed of light, across continents of space. *That hand* that gathers the gentle beauties from nature's lap, to enrich the herbarium of science. *That hand* that culls the sparkling gems from her jeweled laboratory to set in diadems of glory around the scientific arch. But still *that hand*, whose authenticity is denied because, forsooth, its laws of motion cannot be measured, weighed, computed, or even conceived of as an element of power.

In regard to the limit of human investigation, it is only necessary for us to settle clearly in our minds what science is. In the paper before alluded to, it was assumed to be the discovery of laws in nature that by their harmonious relation to each other, and taken in their natural grouping as a whole, gave a partial solution, or philosophy of action. In order to understand the extent of any science it is only necessary to fix in the mind the extent and nature of the things to be considered, and the manner in which the knowledge sought is to be obtained.

We look forth upon the world and system of worlds, and discover how complex and still how perfect are all the constituents and movements, and we feel gratified if we can but grasp the simple outline of laws that their movement presents.

We come down to more tangible objects, and take in our hand comparatively an atom of matter, and we find it just as wonderful in its construction. Sight blurs at the last receding disclosure of perfection, and we seize the microscope and again open another field of beauty, harmony, and perfection, and conclude that its laws of construction are infinite. That "all are parts of one stupendous whole," whose Maker stamped them with his own infinite perfection. In order to see both extent and manner of scientific groupings of laws let us briefly examine mathematics, the *most perfect*, and music, the most pleasing of all sciences.

The science of mathematics although justly considered as fundamentally connected with all the sciences and justly ranks high in the circle, still it is, after all, but the adjunct or private secretary to the other sciences. It is but the accountant. It is what the "field notes" are to the civil engineer. It is what the book-keeper is in a commercial house, and it is exact as far as it goes, because it deals with objects already defined.

Suppose a planter with his lands, his horses and cattle, and other property, wishes to understand fully the extent and quality of his property and products. He studies the anatomy and physiology of his stock, the geological and chemical constituents of his lands, and the botanical structure of his products.

Mathematics is merely the accountant, the numerator of his flocks and herds, with the relation one bears to the other. It has nothing to do with qualities. It is a well known axiom in physics that no two ponderable bodies can occupy the same space at one and the same time. All of the materials with which mathematics deals, must be as perfect axioms furnished to the hand of the mathematician, and all we can say is that this science has proved a very competent book-keeper.

Suppose we express the science of quantity or measurement thus:

Starting from the centre O of this body, we compute the length and breadth, and determining the depths, we ascertain with unerring certainty the quantity contained in the whole. Let this represent the entire globe, and suppose we compute its size, weight and relation one part bears to the other to a fraction. What have we done with all our powers and quantities? Why, simply accomplished the demonstration of the simple unit of one. For this, to us great world, is but one of the myriads within our area of vision even, and could we compute them all, an eternity of bodies and distances lie beyond our mortal ken.

But there are some curious things connected with this science. It can be proved by any mathematician that two lines starting in close proximity to each other shall converge toward a common centre that can never be attained within the limits of calculation, and more, he can demonstrate that they can never reach it.

Again, the phenomena of that peculiar natural development of powers of computation as they are sometimes met with, is yet to be explained.

There are persons who will give the solution of a mathematical problem of the most intricate and extensive computation almost as soon as its terms are stated. These natural mathematicians as they are called, are supposed by some to possess intuitive powers, because some of them at least, are totally unable to tell how they accomplish it.

Will this idea of intuition answer the truly scientific inquiry? The great difference between the animal and man is that man reasons, but the animal is governed by instinctive or a sort of intuitive power. Can it be said that our reasoning powers ever act intuitively?

Is it not probable that the mind, possessing more agility than the hand, (which can be moved imperceptibly,) passes through trains of reasoning, leaving no trace in memory, but that after all, it really does perform all the mental acts necessary to the solution.

There are undoubtedly certain physical conditions into which persons may be thrown by disease or some other cause, where the acuteness of some of the senses are increased to that degree that imperceptible modes of communication are open to them, and because we cannot detect it we call it intuitive or supernatural. To illustrate: There was a gentleman living in St. Louis some years since, who was very susceptible to what is called mesmeric influences. Being a gentleman of undoubted veracity, he afforded a good subject for the careful examination of that phenomenon. Among other things he would, with his eyes securely blindfolded, point to any of his friends on their names being called, although they purposely changed their positions after the bandages were applied. A physician present on one occasion when this feat was performed, quietly slipped off his boots and walked to the other part of the room, and the clue was lost. The patient could no longer detect his location. On being interrogated, he earnestly declared that it was not by sound that he had been guided, but by carefully repeating the experiment he was able to make himself for the first time cognizant of the fact, that his sense of hearing being rendered abnormally acute, he was able to detect his friends by their step. He was, without doubt, unconscious of a power which he used with such unerring certainty. May it not be so with the natural mathematician? May it also not be probable that the mind flies to conclusions by a new path not known to us or science, as now understood? These are points that perhaps are not unworthy a careful consideration. But, it is sufficient to our purpose to know that so long as the field of computation is as vast as eternity itself, no human mind shall ever be satisfied that all is known.

The next science, music, will more clearly illustrate the manner of collecting facts or laws, their grouping into a science, and their necessary imperfections as *forming a perfect science.* As the grammar of a language is derived from the language, and not the language from the grammar. So in music, the science is derived from the language of sound. The language of music is but the *voice* of *pleasure* or *pain.*

It is undoubtedly as universal (in the human family at least) as their capacity to express, in oral sounds, the note of joy or the wail of sorrow.

The human system seems to be largely supplied with countless nerves of sympathy that telegraph to the great centre the first approach of danger or pleasurable appeals from outward sources, but the human soul is as much more extensively supplied with means of communication with external objects and influences, as the mind extends in its power beyond the physical organization. If the body is supplied with a delicate network of nerves, the soul is enshrouded in a perfect halo of living ethereal tissues.

As the trees of the forest sway their huge branches to the wind. As the green leaf'd boughs of humbler growth bend in obeisance to the breathing morn. As the rich verdure that clothes the field and the floral beauties that nestle closer to their mother's genial breast. All nod their heads to the gentlest breeze. So from the richer soil of human hearts, living verdure hangs its clustering tendrils out, all sensitive to the touch of air-laden waves of sympathetic sound.

Though some, like the tall monarchs of the forest, stand defiant to gentle gales, and answer only to the voice of storm and tempest.

Some, like leafless, branchless trunks, lift their rifted forms unmoved by all save the lightning shaft. Some, like creeping, crawling things, hide themselves away from air and light. And some shake silken locks in very glee to every floating zephyr's breath, whose waving tresses are but parasitic graces—borrowed to hide a withered heart.

The earth is not all clothed with things of beauty, but the very contrast renders the great harmonies all the more perfect. So human hearts are not all clothed with the evergreen of sympathy that moves them to the "concord of sweet sounds."

May they too not have their use as in music occasional discordant *flats* and *sharps* are thrown in for contrast. But the science of music, what is the philological relation of this language to the science?

Like other languages, this voice of pleasure or pain is conventional in its meaning, although its cause is spontaneous.

The human family will talk and they will sing, and it is only necessary to arrange their conventional expressions as far and as fast as they progress into a grammatical or scientific system.

A glance at its history will show how far this has been done. The language in which the inhabitants of China and India give expression to their musical proclivities may be expressed thus:

A semitone between the 4th and 5th. The primitive Irish and Scotch gamut is still more singular, thus:

The first semitone occurring between the 4th and 5th, and the second between the 6th and 7th. Their minor scale has but six notes and one semitone, and that between the 2d and 3d, producing the minor 3d.

The Arabs, Turks, Persians and other Oriental nations use a scale of thirds only.

The European major scale, as it is called, is arranged thus:

The minor scale simply places the first semitone between the 2d and 3d, and changes the semitone between the 7th and 8th in the descending scale from that to the 6th and 7th.

All the music with which we are acquainted is executed according to this scale. That is, this scale represents the succession of notes that our ears are accustomed to, and it is not too much to affirm that it is as much a demonstration of progress as the enlightenment, civilization and refinement that has accompanied it.

It is the only scale giving to melody and harmony a tonic and dominant, the only foundation on which melody and harmony are constructed, and the only principle that gives to modulation its vitality. There is another remarkable and significant fact too. It is the only scale that gives to melody or harmony a point of rest or repose.

You may listen to a succession of notes executed according to other scales, and it will create a feeling of unrest. It is like a bird on restless wing. But a single melody, executed according to the European scale, will commence and end at this key-note point of repose. The whole melody as well as harmony is built upon it, and are as truly dependent on it as the arrangement of letters to express thought by words, or the necessity of vowels in their formation.

This key-note or tonic as it is called, is simply the standpoint or home of melody. A single example will perhaps illustrate what is meant. The familiar air of

"Home, sweet home."

Those who are acquainted with the air will be able to follow the words with the air as repeated,

'Mid pleasures and palaces though we may roam,
Be it ever so humble, there's no place like Home,
A charm from the skies seems to hallow us here,
Which, seek through the world, is ne'er met with elsewhere.
Home, Home, sweet, sweet Home,
There's no place like Home, there's no place like Home.

The key-note or tonic represents the simple cottage, the happy home. The spirit, full of its precious associations, bursts forth in an excursive flight to the "*pleasures* and *palaces*," but soon returning, it contrasts the "*ever so humble*" cottage and settles like a bird to its nest, with the feeling that "*there's no place like home.*" From that spot it again soars with the halo that surrounds it as "*a charm from the skies that hallows it*" there, and feels that through the wide world there is no place like it, and as in rapture it hovers over it ere it settles down to rest, repeating

*Home, Home, sweet, sweet Home.* It is not the poetic beauty that is the point presented as the illustration, but the concurrent tone of melody that clings to its twin sister of poetic birth like an angel companion.

This is the true meaning of the vocal adaptation of music to poetry. Poetry vitalized, or simply a higher order of elocution than lies in the power of language, in its common use, to attain.

Let any elocutionist repeat the poetry of "home, sweet home," and by considering his hand at rest as the key-note, and in his gestures, that hand will write the music.

The European scale then will be considered as embracing all of the higher principles of melody and harmony, and as such, the system embodies all there is of the science as it is now understood.

From the fact that music is written on lines and spaces representing a regular succession of degrees, it is supposed by many that these are regular mathematical distances, and that thus far at least, music is reduced to a perfect science, inasmuch as sounds graduated by these distances produce melody, and two or more sounds according to the same scale, produce harmony.

The science of accoustics, too, will attempt to demonstrate that the vibrations of waves of atmosphere produce this harmony by concurrent waves; as for instance, the vibrations of any given note is simply doubled in velocity to produce the accord of an octave higher, or diminished in the same ratio to produce the octave lower—thus:

If this were strictly true, the piano would be as perfect an instrument as the violin.

In order to understand the mistake of such deductions based on mathematical calculations, suppose we tune a piano according to this strict scale of vibration. The result will be an imperfect vibration of about one-ninth of a tone in each additional octave, or a semitone in 4 octaves, or 29 notes, and all the arts resorted to by musicians have hitherto failed to reconcile for practical use this discrepancy, and when it is added that this is but one of the many difficulties to be met with in all attempts at mathematical exactitude, it shows us the science of music to be, as it is, but an approximation to the perfection of nature in the gift.

Is not this a fair representation of the manner in which we acquire and arrange the truths and a just view of human perfectability in the arrangement of any science? Are they not all feeble approximations—the finite tracings of infinite perfection? Before leaving the subject of music there is one feature in it that may not come amiss as a parallel or concedent fact in other sciences. What is called the common ear, (or the natural appreciation which most of us have by nature,) recognizes common chords and a few of the simple harmonious changes in music. But let a person practice these extensively, and they will become monotonous as other beauties falling in one by one as the ear becomes (as we say) educated, until modulations into other keys and partial discords become necessary to the fullest enjoyment, and the music that will delight him most would not be appreciated by the great mass. Is there a doubt that there are persons living in a world of harmony and melody that to us would give no pleasure, simply because we cannot appreciate what are real beauties to the fully educated ear? Science has attempted to lay down rules for harmony and modulation, but some of the greatest composers have built their entire reputations on a perfect departure from those rules, and have held the world, as it were, enthralled by the new development.

Others have lived and died in advance of the age in which they lived.

Who shall judge whether such men are men of science or fanatics? Who shall deny to them the honor of really scientific achievements? Shall the man who can appreciate no harmony above a common chord dare to judge a Handel, a Haydn, or a Mozart?

Is not the rule applicable to other sciences? Shall the man who inhabits the mountain gorge, who from want of will or capacity, circumscribes the boundary of his vision to that narrow space, doubt the assertions of him who has climbed to its highest peak and sees "*Alps on Alps arise?*" Still there are men far up in the mountain acclivities of science who turn their backs to the glowing landscape, and while content to delve in the rocky cliffs, deny that

> **"Though round its breast the rolling clouds are spread,**
> **Eternal sunshine settles on its head."**

The foregoing illustrations are but feeble shadowings of the extent and manner of acquiring scientific knowledge.

It has not been in the design of this paper to exaggerate or to magnify the importance of the one or misstate the errors of the other, but as the empirical reasoner has ever chosen the shield of mere skepticism in philosophy without giving us any other system, or attempting to act as the honest guide or teacher, it has seemed necessary to unmask the errors by placing them side by side with nature's teachings, that they might be contrasted.

Can anything be more plain than the duty of students in science, to open the eye of the mind fully and fairly on the great page on which nature writes her laws? And as he attempts the grouping of them into sciences, to place on its page a full and fair account of all the means used in the compilation and to state the full relation one bears to the other.

One of the first things a child in studying geography has to learn, is to distrust the evidence of his senses, as he is taught that the earth is not flat as he had supposed and by faith in higher teachings he is led to abandon his truly empirical notions for glorious principles founded on higher facts than the senses supply. Are we not very children in the study of nature, and shall we set up the evidence of our physical senses against her higher teachings?

The empirical teacher would take us far away into the twilight solitudes of a frozen zone and point to the splendid ice palaces that lie cold and sharp against a moonless sky, and call that a sample of earth's sublimity, and as the sun in his semi-annual visit just peeps above the horizon and dips again behind the icy battlements, he would point to that as the glorious illuminator of the world. What is science, stripped of the light, beauty and warmth of a living animate philosophy, but a congealed shapeless mass, floating like an iceberg toward a frozen sea. What is science without the light that comes from the great source but a very chaos on which no command of day has ever dawned? But science illuminated is a world smiling with genial warmth and beauty, clothed with a living verdure.

There is a species of popular cant against all kinds of metaphysical reasoning, and the term metaphysics is odious to some who say that it is a system of mere idle speculation. This must be a mistaken view. Metaphysical science is the only expounder of the ethical relations man sustains to the whole circle of moral and physical laws enshrouding his being, and as students of those laws, we owe it to ourselves, we owe it to society and an error-loving world, to give the great lines of truth their due prominence.

It is a settled principle, as shown by the history of idolatrous nations, that they assimilate to the character of the gods they worship. If we are not idolators our attachments all obey the same law of assimilation, and our actions will inevitably rise only to our standard of appreciation.

What says history of the influence of this semi-materialism, this empirical skepticism in the practical philosophy of our social system?

It shows that the *empirical*, *tangible dollar*, occupies a higher place than the true philosophy of its use in ministering to our wants, necessities and charities. It shows that *place* and the gaudy trappings of *position* are the tangible objects sought, instead of the virtues that *alone* give *true dignity*. The empiric, if he attempt the visible worship of any thing, will select the tangible god of self, or content himself with thoughts no higher than adoration of simple rites and ceremonies. Our glorious fathers, who carved out here the most glorious empire of earth, did it by the evolution of a purely metaphysical system of human rights.

The empirical serfs of kingly power, could understand the tangible embodiment of despotic rule, and were like other reasoners, content to stop their reasonings at the throne, paying tribute and devotion to that they could see and understand. But those hero philosophers that planted the "stars and stripes" of an emancipated empire on American soil, achieved the glorious victory by the illumination of a single metaphysical power, and that power stands forth emperor to-day.

## Figures and Tables

**Figure f1:**
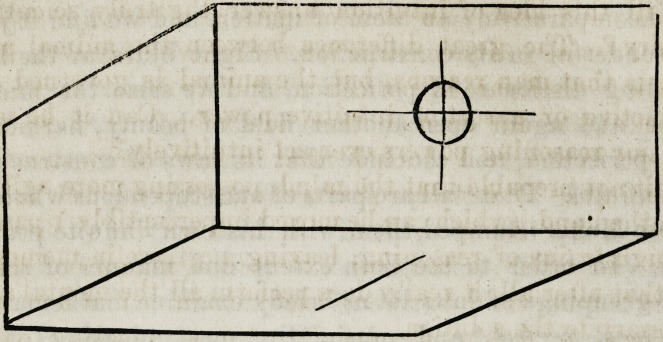


**Figure f2:**
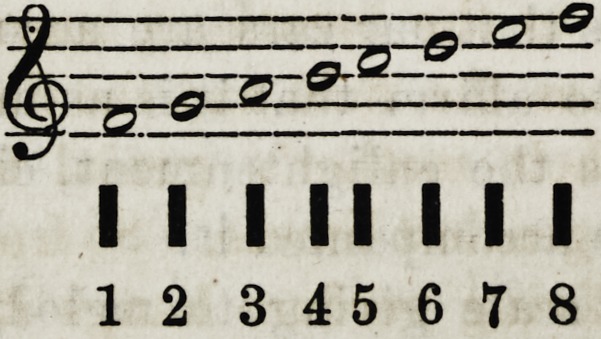


**Figure f3:**
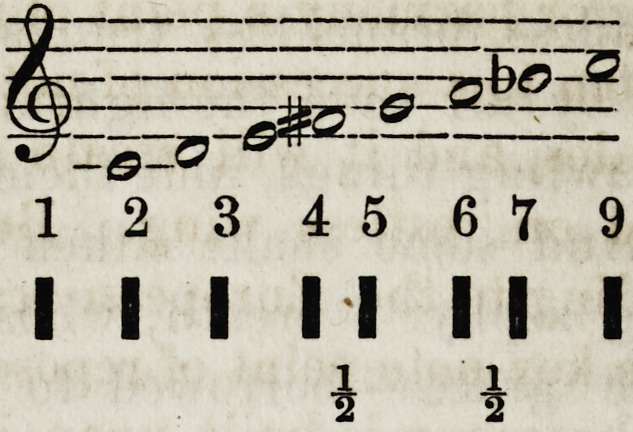


**Figure f4:**
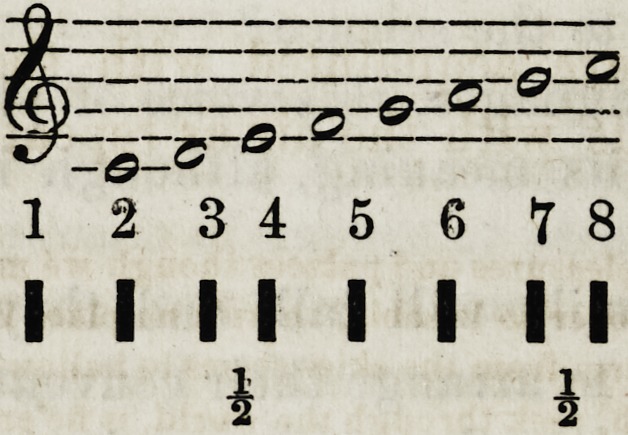


**Figure f5:**